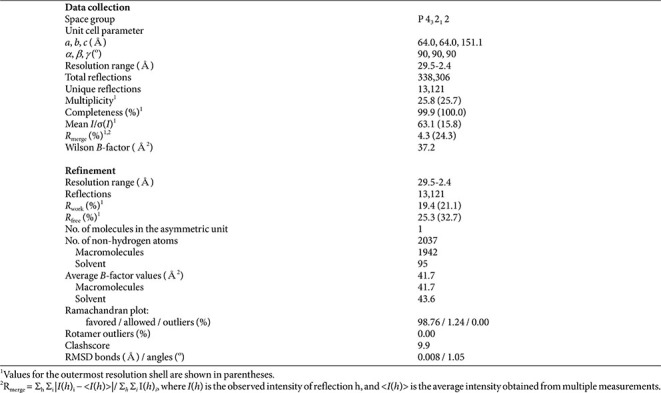# Crystal Structure of Periplasmic Domain of YejM from *Klebsiella pneumoniae*

**DOI:** 10.4014/jmb.2026.02020

**Published:** 2026-04-24

**Authors:** Kyoung Ho Jung, Hyun Ji Ha, Mina Son, Hyun Ho Park

**Affiliations:** College of Pharmacy, Chung-Ang University, Seoul 06974, Republic of Korea

**Keywords:** Crystal structure, *Klebsiella pneumoniae*, YejM, Lipid homeostasis

## Abstract

YejM (also known as PbgA) is an essential inner membrane protein in Gram-negative bacteria that plays a central role in maintaining outer membrane lipid homeostasis. Although the periplasmic domain of YejM has been structurally characterized in *Escherichia coli* and *Salmonella enterica*, structural information from other clinically important pathogens remains limited. Here, we report the crystal structure of the periplasmic domain of YejM from *Klebsiella pneumoniae*. The structure reveals a conserved α/β fold that closely resembles previously reported YejM structures, with strong structural and sequence conservation across Enterobacteriaceae. Comparative analyses demonstrate that key architectural features and conserved residues implicated in lipid sensing are preserved in *K. pneumoniae*, supporting a conserved functional role for YejM across species. These findings extend current structural knowledge of YejM to an important opportunistic pathogen and reinforce the view that the molecular mechanism underlying YejM-mediated regulation of outer membrane lipid homeostasis is evolutionarily conserved among Gram-negative bacteria.

## Introduction

The outer membrane (OM) of Gram-negative bacteria is an essential permeability barrier that protects cells from environmental stresses, antibiotics, and host immune defenses [1, 2]. This asymmetric membrane is characterized by an outer leaflet composed primarily of lipopolysaccharide (LPS) and an inner leaflet enriched in glycerophospholipids, and its integrity relies on the precise coordination of lipid biosynthesis, transport, and assembly pathways [3, 4]. Disruption of OM lipid homeostasis severely compromises bacterial viability and virulence, underscoring the importance of regulatory mechanisms that monitor and balance envelope lipid composition [5].

YejM, also known as PbgA, is a conserved inner membrane protein found broadly among Enterobacteriaceae and has emerged as a key regulator of OM lipid homeostasis [6, 7]. YejM is composed of five N-terminal transmembrane helices anchored in the inner membrane and a large C-terminal periplasmic domain [7]. Early genetic and physiological studies implicated YejM in PhoPQ-regulated outer membrane remodeling and bacterial virulence, particularly in *S. enterica*, where loss of YejM function results in severe defects in membrane integrity and sensitivity to antibiotics [8, 9]. Initial structural and biochemical analyses proposed that YejM functions as a cardiolipin transporter, mediating the transfer of cardiolipin from the inner membrane to the outer membrane via its periplasmic domain [9, 10]. Crystal structures of the isolated periplasmic domain of YejM from *E. coli* and *S. enterica* revealed that this domain adopts an α/β fold resembling members of the arylsulfatase and lipoteichoic acid synthase superfamilies, although lacking the conserved catalytic residues required for enzymatic activity [7,10]. These structures suggested the presence of a large hydrophobic cavity that could accommodate phospholipid substrates, providing a structural basis for the proposed cardiolipin-binding and transport function. Despite these insights, the precise physiological role of the periplasmic domain and the molecular determinants underlying YejM-mediated lipid regulation remained unclear.

More recently, high-resolution structural and functional analyses of full-length YejM from *E. coli* fundamentally revised this model [7]. A landmark study demonstrated that YejM does not function as a cardiolipin transporter, but instead directly senses LPS at the periplasmic leaflet of the inner membrane through a highly conserved lipid A–binding motif. Structural data revealed that the periplasmic domain of YejM acts as a pseudo-hydrolase scaffold that engages lipid A using a dense network of backbone-mediated interactions, thereby linking periplasmic LPS levels to the regulation of LpxC stability via the LapB–FtsH protease complex. This mechanism positions YejM as a central regulator of LPS biosynthesis and outer membrane homeostasis rather than a lipid transporter

Although YejM has been extensively characterized in *E. coli* and *S. enterica*, structural information from other clinically important Enterobacteriaceae remains limited. *Klebsiella pneumoniae* is a major opportunistic pathogen responsible for severe hospital-acquired infections and exhibits remarkable intrinsic and acquired resistance to antibiotics, largely due to the robustness of its outer membrane [11, 12]. Given the central role of YejM in controlling LPS biogenesis and OM integrity, understanding whether the structural architecture and functional mechanisms of YejM are conserved in *K. pneumoniae* is of both fundamental and biomedical importance. In this study, we report the crystal structure of the periplasmic domain of YejM from *K. pneumoniae* (hereafter called kpYejM PD). Structural comparison with previously reported YejM periplasmic domains from *E. coli* and *S. enterica* reveals a striking conservation in overall fold, secondary structure organization, and key residues implicated in lipid recognition. Sequence alignment further demonstrates strong conservation of the periplasmic domain across Enterobacteriaceae, supporting the notion that YejM-mediated sensing of envelope lipids and regulation of LPS homeostasis is a deeply conserved mechanism. Together, our findings extend current structural knowledge of YejM to an important human pathogen and reinforce the view that the function and molecular mechanism of YejM are evolutionarily conserved across Gram-negative bacteria.

## Material and Methods

### Protein Expression and Purification

The gene encoding the periplasmic domain of YejM from *K. pneumoniae* (residues Y246–N586; hereafter referred to as kpYejM PD; GenBank accession number CDO15594.1) was chemically synthesized (Bionics, Republic of Korea) and cloned into the pET28a expression vector (Novagen, USA) via the *NdeI* and *XhoI* restriction sites. The resulting plasmid was transformed into *E. coli* BL21(DE3) cells. A single transformant was cultured overnight in 5 mL of LB medium containing 50 μg/mL kanamycin at 37°C. The overnight culture was diluted 1:200 into 1 L of fresh LB medium supplemented with kanamycin and grown at 37°C until the optical density at 600 nm reached 0.7–0.8. Protein expression was induced by the addition of 0.5 mM IPTG, followed by incubation at 20°C for 18h with shaking.

Cells were collected by centrifugation at 3,500 g for 15 min at 4°C and resuspended in lysis buffer consisting of 20 mM Tris–HCl (pH 8.0), 500 mM NaCl, and 25 mM imidazole. Cell disruption was achieved by sonication on ice, and insoluble debris was removed by centrifugation at 14,000 g for 30 min at 4°C. The clarified supernatant was incubated with Ni–NTA agarose resin (Qiagen) for 2 hours at 4°C, then applied to a gravity-flow column and washed with 60 mL of wash buffer containing 20 mM Tris–HCl (pH 8.0), 500 mM NaCl, and 50 mM imidazole. Bound proteins were eluted using the same buffer supplemented with 250 mM imidazole. Elution fractions were pooled and further purified by size-exclusion chromatography using a Superdex 200 Increase 10/300 GL column (GE Healthcare, USA) equilibrated with 20 mM Tris–HCl (pH 8.0) and 150 mM NaCl. The final protein concentration was adjusted to 16.3 mg/mL, and purity was evaluated by SDS–PAGE.

### Crystallization and Data Collection

Crystallization trials were carried out at 20°C using the hanging-drop vapor diffusion method. Drops were prepared by mixing equal volumes of protein solution and reservoir solution containing 8% PEG 6000, 0.1 M Tris–HCl (pH 8.0), and 0.15 M NaCl, and were equilibrated against 0.4 mL of reservoir solution. Crystals were cryoprotected by soaking in reservoir solution supplemented with 30% (v/v) glycerol prior to flash cooling. X-ray diffraction data were collected at -178°C on beamline BL-5C at the Pohang Accelerator Laboratory (Republic of Korea). Data processing was performed using HKL2000 [13].

### Structure Determination and Analysis

The crystal structure was determined by molecular replacement (MR) using PHASER implemented in the PHENIX suite [14]. Given the recent improvements in the accuracy of AlphaFold3 (AF3) prediction models, we used the AF3-predicted structure as the initial search model for MR. Iterative model building and refinement were conducted using AutoBuild (PHENIX), COOT[15], and phenix.refine [14], with quality validated in MolProbity [16]. All structural images were prepared in PyMOL [17].

### Sequence Alignment

Multiple sequence alignment of YejM homologs was performed using Clustal Omega [18].

## Results

### Structural Characterization of Periplasmic Domain of *K. pneumoniae* YejM

**Fig. 1:**
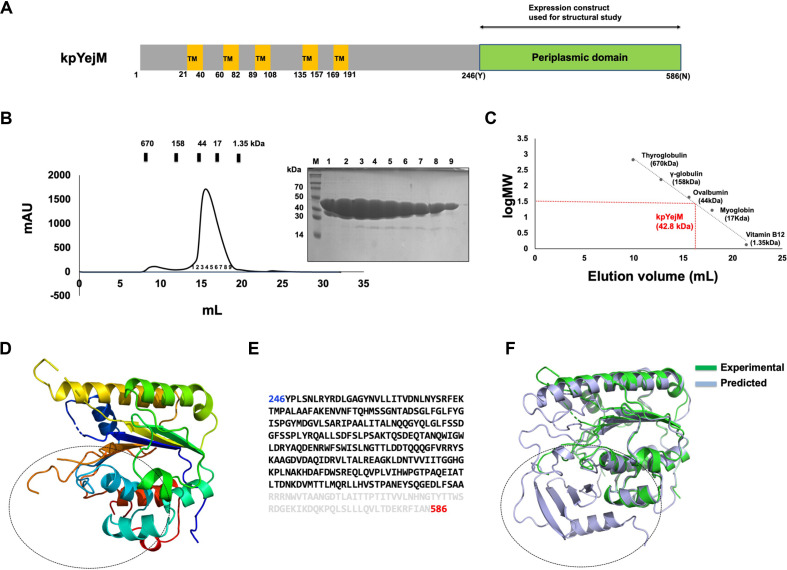
Structural characterization of the periplasmic domain of *K. pneumoniae* YejM (kpYejM PD). (**A**) Schematic representation of full-length YejM showing five N-terminal transmembrane (TM) helices and the C-terminal periplasmic domain. The region used for current structural study (residues 246–586) is highlighted. (**B**) Size-exclusion chromatography (SEC) profile of kpYejM PD. SDS–PAGE analysis of main peak fractions. Numbers above the gel indicate the fraction numbers corresponding to the main peak in the chromatographic profile. (**C**) The major elution peak corresponds to a monomeric species, as indicated by comparison with molecular weight standards. (**D**) Ribbon representation of the crystal structure of the kpYejM PD, colored from N-terminus to C-terminus. The C-terminal region that could not be resolved in the experimentally determined structure is indicated by a black dashed circle. (**E**) Amino acid sequence of kpYejM PD highlighting the C-terminal region (shown in gray) for which electron density could not be traced in the crystal structure but was successfully modeled in the AlphaFold3 prediction. (**F**) Structural superposition of the experimentally determined kpYejM PD structure (green) with the AlphaFold3-predicted model (light blue). The overall fold is well conserved, with minor deviations observed primarily in loop regions. The C-terminal region that could not be resolved in the experimentally determined structure but was modeled in the AlphaFold3 prediction is indicated by a black dashed circle.

Recombinant periplasmic domain of *K. pneumoniae* YejM (kpYejM PD; residues 246–586) was successfully expressed in *E. coli* and purified to homogeneity using Ni–NTA affinity chromatography followed by size-exclusion chromatography (SEC) (Fig. 1A). SDS–PAGE analysis of purified protein showed a single predominant band at approximately 42 kDa, consistent with the calculated molecular weight of the kpYejM PD (Fig. 1B and 1C). SEC analysis revealed a single major elution peak, indicating a monodisperse protein population suitable for crystallization. Calibration with molecular weight standards suggested that kpYejM PD exists predominantly as a monomer in solution (Fig. 1B and 1C).

Crystals of kpYejM PD were obtained using the hanging-drop vapor diffusion method and diffracted to 2.39 Å. The crystal structure was solved by molecular replacement and refined to acceptable crystallographic statistics (Table 1). The asymmetric unit contains a single kpYejM PD molecule (Fig. 1D). The overall fold of kpYejM PD resembles that of the arylsulfatase and lipoteichoic acid synthase superfamilies, characterized by a pseudo-hydrolase scaffold lacking the canonical catalytic residues required for enzymatic activity (Fig. 1D). The domain consists of multiple α-helices and β-strands arranged to form a stable globular architecture, providing a rigid platform for molecular recognition rather than catalysis. Notably, no metal-binding site or catalytic motif typically associated with active sulfatases was observed, consistent with a non-enzymatic regulatory role.

Structural analysis revealed that electron density corresponding to the C-terminal 64 residues could not be reliably traced, precluding model building for this region (Fig. 1D and 1E). Although the absence of electron density for this segment remains unclear, it is likely that this region exhibits substantial conformational flexibility and may adopt multiple conformations depending on the local environment, resulting in poor electron density. To gain structural insight into the unresolved C-terminal region and to assess how accurately it could be predicted, we compared the AlphaFold3-predicted model with the experimentally determined structure of kpYejM PD by structural superposition (Fig. 1F). The comparison revealed minor deviations in several loop regions; however, the overall folding was well preserved. Notably, the C-terminal region that could not be resolved experimentally was well modeled in the predicted structure, indicating that the AlphaFold3 model reliably captures the global architecture of kpYejM PD.

### Sequence and Structure Comparison of YejM Homologs

**Fig. 2:**
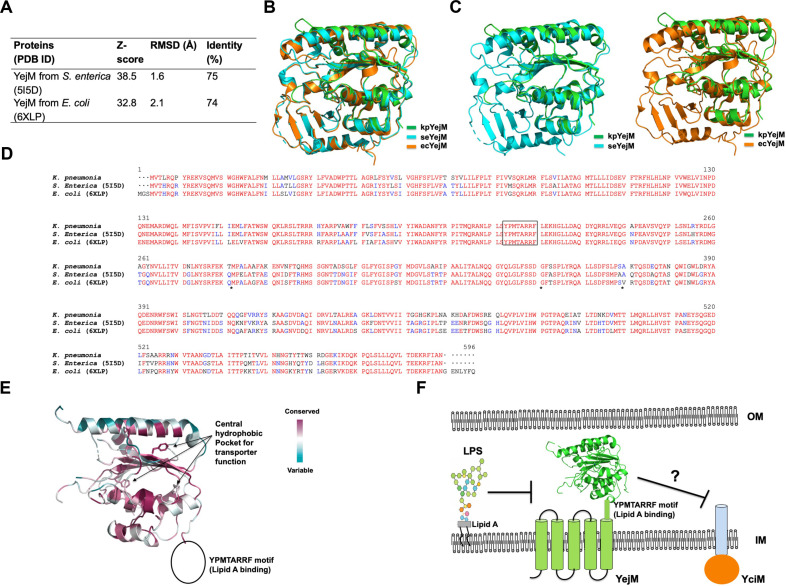
Structural comparison, sequence conservation, and functional model of the kpYejM. (**A**) Table summarizing the result of DALI search. (**B**) Superposition of structural homologous structures shown in cartoon representation. (**C**) Pair-wise structural comparision of kpYejM with seYejM and ecYejM. (**D**) Multiple sequence alignment of kpYejM and its homologs using Clustal Omega. Fully and partially conserved residues are colored red and blue, respectively. In the sequence alignment, the central hydrophobic residues reported to be critical for the transporter function of YejM are marked with asterisks (*). The YPMTARRF motif, known to be essential for Lipid A binding, is highlighted with a black box. (**E**) Cartoon representation of kpYejM mapped with sequence conservation scores calculated by ConSurf server. (**F**) Proposed working model of YejM illustrating lipid interaction and membrane association. YejM is positioned relative to the membrane bilayer to be associated with and working on LPS and YciM to control a membrane homeostasis.

To date, the reported structures of YejM include the periplasmic domain from *S. enterica* (seYejM PD) and the recently determined full-length, membrane-bound YejM from *E. coli* (ecYejM). To examine the structural relationship between kpYejM PD and previously characterized YejM structures, we performed a structural similarity search using the DALI server [19]. As expected, the most structurally similar protein identified was the seYejM PD, followed by ecYejM (Fig. 2A). These results further support the strong structural conservation of YejM across Enterobacteriaceae. The kpYejM PD adopts a compact α/β fold composed of a central mixed β-sheet surrounded by multiple α-helices, consistent with previously reported ecYejM and seYejM structures. Structural superposition of kpYejM PD with homologous YejM PDs revealed a high degree of structural conservation, with low root-mean-square deviation values across the Cα atoms (1.6 Å with seYejM and 2.1 Å with ecYejM) (Fig. 2B and 2C). This conservation highlights the preserved architectural framework of YejM among Enterobacteriaceae. Taken together, the structural data demonstrate that the periplasmic domain of *kp*YejM adopts a highly conserved fold shared among YejM homologs and supports a model in which YejM functions as a structurally conserved regulator of envelope lipid composition rather than as a catalytic enzyme.

In addition to their structural similarity, the amino acid sequences showed a high degree of homology, with approximately 75% identity between homologs (Fig. 2A and 2D). Notably, the central hydrophobic residues (reported to be critical for transporter function) [10] and the YPMTARRF motif, known to be essential for lipid A binding [7, 20], were found to be 100% conserved. (Fig. 2D and 2E). YejM functions as a key regulator of membrane homeostasis, sensing LPS status and modulating its activity accordingly. By antagonizing YciM, which negatively regulates LPS biosynthesis, YejM helps fine-tune LPS levels and maintain outer membrane stability (Fig. 2F). Although the inhibitory mechanism of YejM activity through its binding to LPS has been recently elucidated [7], the mechanism by which YejM suppresses YciM remains unclear. Further studies will be required to uncover the molecular basis of this regulation.

## Discussion

In this study, we determined the crystal structure of the periplasmic domain of YejM from *K. pneumoniae* (kpYejM PD) and performed comprehensive structural and sequence comparisons with previously characterized homologs from *E. coli* and *S. enterica*. Our findings demonstrate that kpYejM PD adopts a highly conserved α/β pseudo-hydrolase fold that is structurally similar to seYejM and ecYejM, with low RMSD values and strong conservation of overall architecture. These observations reinforce the notion that YejM functions as a structurally conserved regulator of outer membrane lipid homeostasis across Enterobacteriaceae.

Sequence analysis further revealed approximately 75% identity among homologs, indicating substantial evolutionary conservation. Notably, residues forming the central hydrophobic pocket—previously reported to be important for transporter-related function—as well as the YPMTARRF motif implicated in lipid A binding, were found to be 100% conserved. The strict conservation of these residues strongly suggests that they play critical functional roles. Given that recent studies have demonstrated that YejM senses LPS via direct lipid A binding rather than functioning as a cardiolipin transporter, the preserved hydrophobic cavity and conserved lipid A–binding motif likely represent key structural elements required for LPS recognition and membrane homeostasis regulation.

Among homologs, the remarkable preservation of both structural framework and sequence features suggests strong evolutionary pressure to maintain YejM function. Such high conservation, particularly within functional motifs and hydrophobic core regions, is consistent with the essential role of YejM in regulating LPS biosynthesis and outer membrane integrity. Because disruption of LPS homeostasis severely compromises bacterial viability, it is plausible that these conserved structural elements are indispensable for maintaining envelope stability in Gram-negative pathogens.

Interestingly, in the kpYejM PD structure, the C-terminal region could not be resolved due to lack of electron density, suggesting structural disorder or substantial flexibility. In contrast, the corresponding region is well resolved in the previously reported ecYejM and seYejM structures, indicating a more ordered conformation under their respective experimental conditions. This discrepancy suggests that the C-terminal region of YejM may exhibit species-dependent conformational variability or be sensitive to environmental or crystallization conditions. Consistent with this idea, the AlphaFold3-predicted model presents this region in a defined conformation, implying that it may adopt a structured state under specific physiological conditions. Such flexibility could have important functional implications, as dynamic regions are often involved in regulatory interactions, conformational switching, or protein–protein binding. Accordingly, the flexible C-terminus of kpYejM PD may contribute to modulating interactions with regulatory partners, such as YciM or other periplasmic factors involved in LPS regulation. Further biochemical and structural investigations will be required to determine whether this region undergoes conformational changes upon ligand binding or interaction with regulatory complexes.

Although the mechanism by which LPS binding inhibits YejM activity has recently been elucidated [7], the molecular details underlying YejM-mediated suppression of YciM remain unresolved. Given the structural conservation observed in kpYejM PD, it is reasonable to propose that the regulatory mechanism identified in *E. coli* is likely conserved in *K. pneumoniae*. However, species-specific regulatory nuances cannot be excluded, particularly considering differences in pathogenic lifestyle and membrane stress responses. Future studies focusing on full-length kpYejM in a membrane context, as well as interaction analyses with YciM and associated proteolytic machinery, will be necessary to clarify these mechanisms.

In conclusion, our structural characterization of kpYejM PD extends the structural repertoire of YejM to an important opportunistic pathogen and confirms that the molecular architecture underlying YejM-mediated regulation of LPS homeostasis is highly conserved. The strict conservation of key functional residues highlights their essential role in lipid sensing and membrane regulation. These findings provide a structural framework for understanding YejM function in *K. pneumoniae* and may facilitate the development of therapeutic strategies targeting envelope lipid homeostasis in multidrug-resistant Gram-negative bacteria.

### Accession Numbers

Atomic coordinates and structure factors for the reported crystal structures have been deposited with the Protein Data Bank under accession code 23KZ.

## Figures and Tables

**Table 1:** Data collection and refinement statistics